# The “autopsy” enigma: etymology, related terms and unambiguous alternatives

**DOI:** 10.1007/s12024-023-00729-9

**Published:** 2023-10-25

**Authors:** Jacob Foster

**Affiliations:** School of Medicine, Cardiff University, Cardiff, Wales UK

**Keywords:** Autopsy, Bereavement, Communication, Terminology, Semantics, Linguistics

## Abstract

The concerted use of Greek-derived medical terms in the present day allows us to facilitate effective communication while honouring the historic roots of Western medicine. The word *autopsy* derives from its third century B.C. Hellenistic Greek etymon *αὐτοψία* (“to see for oneself”), later borrowed into Neo-Latin as *autopsia* and Middle French as *autopsie*. Throughout its etymological journey, *autopsie* underwent semantic narrowing from the passive sense “self-inspection of something without touching”, to a purposeful action by an operator performing “an examination of the human body itself”, to specifically “dissection of a dead human body”. These curious turning points for the meaning of *autopsie* produced an auto-antonym: the same word now has multiple meanings, of which one is the reverse of another. The French *autopsie* used in the latter sense predates that documented for the English *autopsy* (attested 1829). Since the early nineteenth century, attempts were made to remedy the discrepancy between conflicting senses either by adding determining adjectives to the existing noun, or by substituting it with another word altogether. This review explores the etymological journey of *autopsy*, considers which related terms have been popularised throughout history, introduces the concept of lexical ambiguity and suggests unambiguous English compound (*necropsy* and *necrotomy*) and Latin-derived (*non-invasive* and *invasive postmortem examination*) alternatives to satisfy a recent appetite for clarity in international professional and next-of-kin communication.

## Introduction

Three quarters of contemporary English medical terminology is estimated to be of Greek origin; unsurprising, given the pioneering impact on modern medicine from 500 B.C. classical Greece [1]. Until relatively recently, linguistic contact between living Greek and English languages was not possible, and so lexical diffusion was necessarily indirect. Vocabulary items were mostly borrowed through Latin, via written media and daughter languages (the Romance languages, particularly French), or from Ancient Greek texts. The concerted use of Greek-derived medical terms in the present day allows us to facilitate effective communication while honouring the historic roots of Western medicine.

One such medical term now more commonly represents a procedure that directly contradicts its original intended sense. As a result, the word *autopsy* has, throughout history, bewildered death investigation stakeholders. Its continued use in the decision-making process for how invasive a *postmortem examination* ought to be may confuse and alienate families at a time where clarity is exceptionally important. How are we meant to counsel and consent the deceased’s next-of-kin if we, as death investigators, cannot agree on definitions for the very procedures we are proposing? This review explores the etymological journey of *autopsy*, considers which related terms have been popularised throughout history, introduces the concept of lexical ambiguity, and suggests unambiguous alternatives to satisfy a recent appetite for clarity in international professional and next-of-kin communication, as discussed by previous authors [2–5].

## Etymology and semantic change

The term *autopsy* derives from its third century B.C. Hellenistic Greek etymon *αὐτοψία* (*autopsia*, “to see for oneself”); an amalgamation of *αὐτός* (*autos*, “oneself”) and *ὄψις* (*opsis*, “sight; view”) [6]. *Αὐτοψία* at this time vaguely denoted the self-inspection of something, without physically touching it. The object being inspected or observed could be virtually anything, and was certainly not restricted to deceased human bodies. It was used in a literal sense to portray self-inspection by Galen (*Κλαύδιος Γαληνός*; 129–216 A.D.) in his seminal text, later translated into the Latin *De Anatomicis Administrationibus* [7]. The Byzantine Greek *αὔτοπτος* was used until 1453 and subsequently borrowed into Neo-Latin as *autopsia* [6]. *Autopsia* came to reference those observations made on live patients by a physician for the purposes of diagnosis, contrasting with *historia* (denoting information supplied by patients themselves) [8]. It was much later when the phrase *autopsia cadaverum* (“autopsy of cadavers”, with variants like *autopsia cadaverica*) was written into several Latin medical texts, including the 1765 *Synopsis Universae Praxeos-Medicae* of the French physician Joseph Lieutaud [9].

*Autopsia* transitioned into the Middle French *autopsie*; attested 1573 from a source cited in Desmaze’s *Curiosités des anciennes justices* (though the context does not make the precise sense clear) [10]. *Autopsie* is again attested 1665, without context, in a list of scientific terms used in the unpublished letters of a seventeenth century French physician [11]. Authoritative dictionaries have assigned these instances to the sense “postmortem examination” [6]. However, given the lack of source context, widespread religious prohibition to human dissection pre-eighteenth century, and the infrequency with which the sense “postmortem examination” was referenced at the time, it seems probable that in at least one of these two instances the author(s) meant “careful visual examination of a living patient”. The French *autopsie* underwent semantic narrowing from the passive “self-inspection of something without touching”, to a purposeful action by an operator performing “an examination of the human body itself”, to specifically “dissection of a dead human body” [12]. This curious turning point for the meaning of *autopsie* created an auto-antonym: the same word now has multiple meanings, of which one is the reverse of another. The French *autopsie* used in the latter sense predates that documented for the English *autopsy*, Spanish *autopsia*, Italian *autopsia* and German *autopsie*; although attestations are rare in all languages before the beginning of the nineteenth century [11]. Perhaps as a result of the lexical ambiguity of *autopsie*, attempts were made to remedy the discrepancy between conflicting senses either by adding a determining adjective to the existing noun (the popular *autopsie cadavérique* is attested 1801, and the rarer *autopsie cadavéreuse* 1821), or by creating the newer *nécropsie* to specifically denote “an examination of a corpse” (attested 1826). However, the latter has never succeeded in supplanting *autopsie* [11, 13].

Use of the English *autopsy* as applied specifically to “an examination of a dead human body” is attested 1829, when von Ruhl, Creighton and Bluhm made an account of the case of the Empress Feodorovna of Russia [7]. The term was accepted by 1881, at which point the *New Sydenham Society’s Lexicon* for that year reads “it has of late been used to signify the dissection of a dead body” [14]. In the same text, *autopsy* appears alongside *autopsia* (“self-inspection; evidence actually present to the eye”) and the elaborative *autopsia cadaverica* (“a post-mortem examination”). Pepper’s 1949 *Medical Etymology* describes *autopsy* aptly as “a curious term” [8]. The current *autopsy* definition varies according to the source. It can be a noun (i.e. the examination *process*), a transitive verb (i.e. the examination *act*) or an adjective (i.e. *describing* someone or something that has undergone an *autopsy*). The following are excerpts from nine authoritative English dictionaries, defining the former word class:

au●top●sy, noun. ˈɔː.tɒp.si.

**The American Heritage Dictionary of the English Language** [15]:

1. Examination of a cadaver to determine or confirm the cause of death.
2. A critical assessment or examination after the fact.

**Cambridge Advanced Learner’s Dictionary** [16]:

1. The cutting open and examination of a dead body in order to discover the cause of death.

**The Chambers Dictionary** [17]:

1. A postmortem.
2. Any dissection and analysis.

**Collins English Dictionary** [18]:

1. Dissection and examination of a dead body to determine the cause of death.
2. An eyewitness observation.
3. Any critical analysis.

**Longman Dictionary of Contemporary English** [19]:

1. An examination of a dead body to discover the cause of death.

**Macmillan Dictionary** [20]:

1. A medical examination of a dead person’s body to find out why they died.

**The Merriam-Webster Dictionary** [21]:

1. An examination of a body after death to determine the cause of death or the character and extent of changes produced by disease.
2. A critical examination, evaluation, or assessment or someone or something past.

**Oxford English Dictionary** [6]:

- The action or process of seeing with one’s own eyes; personal observation, inspection, or experience.
- Examination of the organs of a dead body in order to determine the cause of death, nature and extent of disease, result of treatment, etc.; a post-mortem examination; an instance of this.
- A critical examination or dissection of a subject or work.

**Random House Kernerman Webster’s College Dictionary** [22]:

1. The inspection and dissection of a body after death, as for the determination of the cause of death.
2. A critical analysis of something after it has taken place or been completed.

As is exemplified above, some lexicographers attempt to capture a physical act with phrases like “examination of the organs” and “cutting open”, while others fixate on the outcome: “to determine the cause of death” or “changes produced by disease” [2]. These definitions would infer that the primary aim of the *autopsy* is to determine the cause of death, and there is no mention as to how this might be achieved apart from cutting or dissecting. None of the aforementioned definitions for *autopsy* represent fully the diversity of postmortem procedures for the purposes of death investigation. For instance, the *postmortem examination* does not necessarily involve entering the body in any way, and its aim is not always to find a cause of death either: amongst other things, they help to determine viability in infants, manner of death and post-mortem interval; they facilitate identification and organ retrieval; and can be used for research purposes. In short, one might make a *postmortem examination* of varying invasiveness in order to answer several different questions from a range of stakeholders.

Forensic pathology texts use the word *autopsy* frequently, some exclusively, with authors providing their own definitions. Knight refers to the *autopsy* as “an innately destructive process [that] can cause artifacts”; Dolinak writes “the *autopsy* consists of an external examination, followed by internal examination of the organs”; and Prahlow describes “a surgical examination performed on a dead body… involves opening the abdomen, chest, and head to examine and then remove the organs for dissection, with or without subsequent examination of microscopic sections” [23–25]. The Human Tissue Authority, National Health Service and Royal College of Pathologists all define *autopsy* vaguely as “an examination of a body after death” [26–28]. In contrast to the English interpretation of *autopsy*, Greek forensic practitioners use their translated equivalent *αυτοψία* to refer to any careful examination, without destroying evidence, of the crime or death scene [3]. This interpretation is a more literal one; a testament to the relatively direct evolution from Ancient to Modern Greek language.

## Related nouns and determining adjectives

**Fig. 1:**
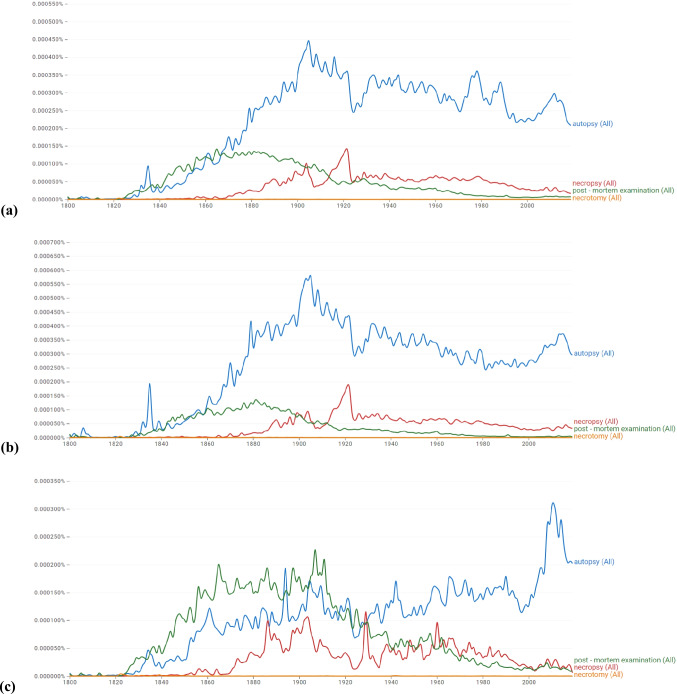
Google Books Ngram Viewer graphs showing how frequently the words *autopsy*, *necropsy*, *post-mortem examination* and *necrotomy* occurred in a corpus of books from 1800 to 2019 in: **a** English published in any country; **b** English published in the USA; and **c** English published in the UK [29]

Nowadays, *autopsy* occurs between 1 and 10 times per million words in typical modern English usage, along with other words which are considered to be distinctively educated, while not being overly technical or jargon (example nouns at a similar frequency include *surveillance*, *assimilation* and *paraphrase*) [29]. Since the early nineteenth century, attempts have been made to remedy the discrepancy between conflicting senses either by adding determining adjectives to the existing noun, or by substituting *autopsy* with another word altogether, although none have succeeded in surpassing its popularity for over a century (Fig. 1).

The term *postmortem examination* is an example: a borrowing from Classical Latin *post* (“after”) and *mortem*, accusative of *mors* (“death”), attested 1834 [30]. The term is frequently shortened simply to *postmortem*, and may be hyphenated or unhyphenated for the sense “examination of a dead body” (although the latter is not also used for the “after death” adverb form). Knight remarks “the term ‘*post-mortem examination’* is a common alternative, especially in Britain, where its meaning is never in doubt. Unfortunately, it suffers from a lack of precision about the extent of the examination, for in some countries many bodies are disposed of after external examination without dissection” [23]. However, one may argue that the word *autopsy* provides even less information about the content of the examination, given its original sense “self-inspection of something without touching it” and current polysemy. Knight observed the relative popularity of *postmortem examination* over *autopsy* in Britain; use of the former was preferred between the 1830s and 1930s in British English compared with American English texts, as represented by Fig. 1. Substitutions of *autopsy* for *postmortem examination* were common: the 1885 English translation of Virchow’s *Die Sections-Technik* preferred the term *postmortem examination* over *autopsy*, and similarly Hektoen in his 1894 *The Technique of Post-mortem Examination* [31]. Nowadays in the United Kingdom, statutory and regulatory bodies tend to either offer vague, overarching definitions for *autopsy*, or replace it altogether with *postmortem examination*, as has been the case with recently amended Home Office publications [32]. UK Government legislation makes no reference to the *autopsy*, and instead refers only to *postmortem examinations*. This is epitomised by Acts governing activities involving human tissue [33, 34], and those involving the authorisation of *postmortem examinations* by judicial officers [35, 36].

A contributor to JAMA’s 23^rd^ issue in 1901 poses a dilemma presented to the US Circuit Court in Kentucky, illustrating the importance of accurate language in these circumstances [37]: when a person taking out a life insurance policy permits a medical advisor to *examine* the body after death, does this give the company the right to make an *invasive postmortem examination*? Indeed, the court “did not think that any ordinary person would suppose that they were agreeing to what would have been much more clearly expressed by the word ‘*autopsy*’ or by the word ‘*dissect*’… While an *autopsy*, generally speaking, always includes an *examination*, the court does not think that an *examination* always includes an *autopsy*”.

Another term that overtook *postmortem examination* in popularity from the 1910s was *necropsy* (attested 1842), which was formed in English by compounding *necro-* (“death”) and *-opsy* (“visual inspection”); probably modelled on the aforementioned French *nécropsie* [38]. Pepper’s *Medical Etymology* describes *necropsy* simply as “a better term than *autopsy*” [8]. Knight writes “though ‘*necropsy*’ is semantically the most accurate description of the investigative dissection of a dead body, the word ‘*autopsy*’ is used so extensively that there is now no ambiguity about its meaning” [23]. *Necropsy* is also considered a more general term without reference to species [5]. *Autopsy* in its early sense “self-inspection” led many to believe that the frame of reference for “self” was “ourselves”; i.e. our own species, humans. As such, the *postmortem examination* of a non-human was proscribed from using the term and instead designated a *necropsy*. However, the current meaning of *necropsy* is subject to similar criticism as *autopsy*: strictly, the word portrays “inspection of a dead body”, but is more often used in the context “dissection of a dead body”. In contrast to its English interpretation, Greek forensic practitioners use their *νεκροψία* to denote an observation of the intact (not yet dissected) deceased [3]. In Greece, the *necropsy* would be considered synonymous with the *non-invasive* or *external-only postmortem examination*. *Necrotomy* is a compound of *necro-* (“death”) and *-otomy* (“dissection”), and is seldom used in English [39]. The Greek equivalent *νεκροτομία* is used to denote “dissection of a dead body”, and is considered synonymous with the *invasive* or *internal* *postmortem examination* [3].

Several other modern words now use the *autopsy* root to describe various forms of *postmortem examination*, and their quantity reflects the sheer variability in procedures. The least invasive is the so-called *verbal autopsy* (“a method used to ascertain the cause of a death based on an interview with next of kin or other caregivers”); a juxtaposition, given that no examination of the body is actually undertaken, and which Burton suggests would be better represented by *postmortem clinical case review* [40, 41]. Pathological examinations have embraced new technologies, and *non-invasive postmortem examinations* are often supplemented with various imaging modalities. The so-called *virtopsy* is a portmanteau of *virtual* and *autopsy*, and is a trademark registered to Dirnhofer; the former head of the Institute of Forensic Medicine at the University of Bern, Switzerland [42]. A similar buzzword *echopsy* describes a modified *needle autopsy* technique with ultrasonography [43]. Where a *postmortem examination* does not provide a satisfactory answer for the cause of death, the term *negative autopsy* is sometimes used. The use of genetic analytic techniques to determine the cause of death in these unexplained cases is represented by the term *molecular autopsy*; first proposed 20 years ago [44].

Indications for postmortem procedures also vary. In England and Wales, there are two fundamental types of *postmortem examination*: *hospital* and *coronial* (usually subdivided into *routine coronial* and *forensic* cases). The *hospital invasive postmortem examination* rate was 0.51% of all deaths in England and 0.65% of all deaths in Wales in 2013 [45]. Routine *coronial* and *forensic invasive postmortem examinations* were performed in 16% and 0.8% of deaths in the same year, respectively [46]. Confusingly, the vast majority of *postmortem examinations* instructed by the coroner are performed in a hospital mortuary by histopathologists who are also employed by the National Health Service. The term *coronial* strictly means “relating to a coroner”, and therefore any *postmortem examination* authorised by a coroner is, in essence, *coronial*. However, in England and Wales, *coronial* cases tend to refer to those that are not *forensic*. The word *forensic* derives from Classical Latin *forēnsis* (“of or belonging to the Forum; of or connected with the law courts”) and its current definition has largely retained this meaning (“of, relating to, or associated with proceedings in a court of law”) [47]. According to this definition, one would expect the *forensic postmortem examination* to automatically describe *any* qualifying coroner-requested procedure, as is the case in almost every other country with an established forensic pathology service, including Scotland (the Procurator Fiscal distinguishes between those cases likely to progress to court and those not, named according to the statutory requirement for corroboration in Scots law: *one-doctor* or *two-doctor postmortem examinations*) [48]. In England and Wales, the *routine coronial* and *forensic postmortem examinations* are distinguished by the cost to the coroner, requirement for a Home Office registered forensic pathologist to perform the procedure, and a higher level of scrutiny with the expectation that the case will be heard in court.

To complicate things further, *hospital postmortem examinations* are sometimes referred to as *consented*, and their *coronial* counterpart as *non-consented*, given that informed consent is not mandatory in *coronial* cases. However, families must be notified and will likely be counselled on the advantages and disadvantages of a *postmortem examination* as applied to an individual case, and may be asked for their “consent” in the sense that the coroner should pay appropriate respect to families’ held religious and cultural wishes with regards to the treatment of the deceased body.

## Lexical ambiguity and unambiguous alternatives

When deciding how to deploy language in daily conversation or written literature, a decision must be made: is accurate communication more important than ease or tradition? Should we honour words that are common but misleading? An estimated 80% of common English words have multiple related dictionary senses, but the word *autopsy* is antilogous: it represents multiple senses, at least one of which (“self-inspection”) is almost the reverse of another (“dissection of a dead body”) [49]. Because of this, a reader/listener must first decipher exactly which definition is intended to understand any sentence containing the word. This “disambiguation” process involves encountering an ambiguous word, rapidly and automatically retrieving in parallel all known meanings (“exhaustive access”), and then selecting the single meaning that is most likely to fit with that particular context [49]. The most comprehensively-studied and best understood brain regions responsible for this process are the posterior and middle subdivisions of the left inferior frontal gyrus (eponymous “Broca’s Area”) [50]. For words with multiple senses, there may either be a so-called "ambiguity advantage" (ambiguous words with multiple *related* senses are quickly and accurately accessible, conferring faster visual lexical decisions when compared with unambiguous words) or an “ambiguity disadvantage” (multiple *unrelated* meanings lead to slower visual lexical decisions in the same experiments) [51]. At present, there are no published studies investigating which term denoting human dissection is easiest to contextualise, and whether the word *autopsy* confers an “ambiguity advantage” or “disadvantage” relative to its counterparts.

The widespread use of ambiguous language when referring to postmortem procedures will likely lead to skewed perceptions of the general public towards them. The most common sources of *postmortem examination*-related information in the UK are television and mainstream media, so the beliefs held by the public are perhaps unsurprising: 97% of people in a Sheffield-based sample believed that "*post-mortems*" involved “examining the inside of the body” whereas only 84% acknowledged that they involved “examining the outside of the body”, demonstrating a relative ignorance to less-invasive techniques [52]. Recent studies have highlighted the contribution of recent exposure on disambiguation, demonstrating that we are biased to select recently-encountered meanings [53]. So, while the word *autopsy* may strictly refer to *any postmortem examination* (ranging from inspection to dissection), this principle of “word-meaning priming” means that, because the general public are exposed to the word *autopsy* in the sense “dissecting a dead body” more than “inspecting a dead body” from television or media, they may be more likely to favour the more invasive meaning in any given situation.

Instead of using the *autopsy* noun with *hospital*, *coronial* and *forensic* adjectives, it is perhaps more useful for families to define a procedure by: (i) who requested the *postmortem examination*, (ii) for what purpose, and (iii) who intends to perform the *postmortem examination*. For instance, “a *non-invasive postmortem examination* and computed tomography scan requested by a coroner to determine a cause of death, performed by a Home Office registered forensic pathologist” or “an *invasive postmortem examination* requested by a consultant cardiothoracic surgeon to understand the pathophysiology of known surgical complications, performed by a histopathologist”. The definitions in Table 1 would preserve tradition *and* communication by offering a more logical, sensible lexicon for pathologists performing postmortem procedures, and normalise using universally understood language for bereaved families.

**Table 1:** English compound terms, Latin-derived terms, modern synonyms and definitions for various *postmortem examinations* [38, 39]

**English compound term**	**Latin-derived term**	**Modern synonyms**	**Definition**
Necropsy	Non-invasive postmortem examination	External-only postmortem examination; postmortem inspection; view and grant	Inspection of a dead body
Necrotomy	Invasive postmortem examination	Internal postmortem examination; postmortem dissection	Dissection of a dead body

## Language standardisation and implications

Language standardisation is the process by which conventional forms of a language are established and maintained [54]. A standard language typically arises either: (i) without formal government intervention, as is the case with Standard English; or (ii) after being formally prescribed by language authorities, such as the French *Académie Française* and Spanish *Real Academia Española*. Given the poor standardisation of English words denoting postmortem procedures (particularly across state and private dictionaries, forensic pathology texts, and individual institutions), a degree of language planning may be necessary to improve communication.

Language planning in this context, amongst other factors, involves balancing lexical ambiguity, word familiarity, frequency of use, similarity with other languages and tradition. The apparent success of codification depends largely on its acceptance by a population as well as its implementation by Government and authoritative bodies. The term *postmortem examination* is already preferentially used in key UK legislation relating to death investigation and human tissue handling. For pathologists, the proposed lexicon (Table 1) may be used in reports, during court proceedings, and in communications with lay-people and experts alike. For researchers, standard terms may be used in published material, so as to reduce uncertainty about the scope and extent of postmortem procedures, and to facilitate research communication globally.

## Conclusion

The word *autopsy* evolved from its Hellenistic Greek etymon *αὐτοψία* (“to see for oneself”), and progressed through its Neo-Latin and French forms: *autopsia* and *autopsie*, respectively. Only relatively recently has the English word been attributed to the sense “dissection of a dead body”, and since this time it has confounded lay and professional understandings of postmortem investigative procedures. Those working within the death investigation sphere should be aware of the uncertainties surrounding this confusing terminology, and use appropriate, accurate language to describe the procedures they are counselling and consenting families on. The historical and geographical variability of *autopsy* also makes the term unsuitable for communication on an international stage. There have been conscious efforts by policymakers and death investigators to replace the term with unambiguous English compound (*necropsy* and *necrotomy*) and Latin-derived (*non-invasive* and *invasive postmortem examination*) alternatives to satisfy a recent appetite for clarity in international professional and next-of-kin communication.

## Key points

1. The word *autopsy* underwent significant semantic change over the course of history.
2. Modern definitions of *autopsy* are greatly variable, and differ from its original sense.
3. *Autopsy* definitions misrepresent the diversity of postmortem procedures, such that alternative nouns and determining adjectives are needed for clarity.
4. There have been efforts to replace the term with unambiguous alternatives.
5. Using standard language improves international professional and next-of-kin communication.
